# Supplementary material for: Are carbon emissions trading and green financial instruments synergistic? -Comprehensive quantitative research based on content analysis
Source: PLoS One. 2024 Mar 7;19(3):e0298601. doi: 10.1371/journal.pone.0298601 (PMC10919601; doi:10.1371/journal.pone.0298601)
Supplement: S1 Data — (ZIP) [file pone.0298601.s001.zip › Data/地级市Z_F工作报告.zip!/╒π╜¡/─■▓¿╩╨/└┤╫╘╣1⁄2╓┌║┼í╛┬φ┐╦╩2╛▌═°í┐_2018.docx]

各位代表：

　　现在，我代表宁波市人民政府向大会报告工作，请予审议，并请市政协委员和其他列席人员提出意见。

　　一、2017年工作回顾

　　2017年是本届政府的开局之年。一年来，面对错综复杂的国内外形势和艰巨繁重的改革发展稳定任务，我们在省委、省政府和中共宁波市委的正确领导下，认真学习贯彻习近平新时代中国特色社会主义思想，深入贯彻落实党的十八大、十八届历次全会和十九大精神，坚定不移实施“八八战略”，按照省市党代会的决策部署，牢牢把握稳中求进工作总基调，牢固树立新发展理念，坚持以供给侧结构性改革为主线，凝心聚力，攻坚克难，统筹推进各项工作，实现了“名城名都”建设的良好开局。

　　一年来，全市经济社会平稳健康发展，多数指标好于市人代会确定的目标，民生实事项目全面完成。初步核算，实现地区生产总值9846.9亿元，按可比价计算，同比增长7.8%；完成一般公共预算收入1245.3亿元，增长10.9%；完成固定资产投资5009.6亿元，增长3.5%，其中民间投资增长11.9%；外贸进出口总额7600.6亿元，增长21.3%，实际利用外资40.3亿美元；社会消费品零售总额4047.8亿元，增长10.4%；城乡居民人均可支配收入分别为55656元和30871元，增长7.9%和8%。一些重点工作取得突破性进展，宁波“一带一路”建设综合试验区获省政府批复，“16+1”经贸合作示范区列入《中国—中东欧国家合作布达佩斯纲要》，成为全国第九个累计实际利用外资超500亿美元的城市，宁波舟山港成为全球首个货物吞吐量超10亿吨的港口，宁波大学入选国家一流学科建设高校，荣获全国文明城市“五连冠”，第八次获评“中国最具幸福感城市”。

　　一年来，我们围绕市人代会确定的目标和“十个狠抓”的任务，全力以赴抓落实，奋发奋进求实效。

　　（一）实体经济发展得到新提升。工业经济再上新台阶。规上工业增加值、利税分别超过3000亿元、2000亿元，增长9.6%和22%。推进“中国制造2025”试点示范城市建设，打造“3511”新型产业体系，“3+15+X”政策框架体系基本形成，市本级财政统筹安排三年150亿元扶持资金。新材料、高端装备、新一代信息技术等三大战略引领产业产值增长27.9%，汽车制造、纺织服装等五大优势产业产值增长20%。千亿级、行业骨干、高成长等三类培育企业产值分别增长31%、24%和37%，新增全国企业（产品）单项冠军15个。攻坚推进100个重大项目，完成投资1807亿元。舜宇智能光电等项目建成投产，吉利汽车整车生产等项目稳步推进。新增上市公司20家、“新三板”挂牌企业31家，宁波股权交易中心挂牌企业突破1000家。现代服务业提质发展。实现服务业增加值4427.3亿元，增长8.1%。金融业稳步提升，人民币贷款余额超过1.7万亿元，新增各类金融机构25家。国家保险创新综合试验区建设成效显现，中国保监会14条支持政策落地实施，全省首家保险资产管理公司获批设立，新增保险创新项目29个。港航物流业发展迅速，国家综合运输服务示范城市建设深入推进，宁波舟山港完成集装箱吞吐量2460.7万标箱，增长14.1%，海铁联运箱量增长59.9%，机场旅客吞吐量939万人次、货邮吞吐量12万吨，快递业务处理量突破10亿件。

　　旅游业蓬勃发展，国内外游客突破1亿人次，获评中国旅游休闲示范城市。新兴服务业加快培育，商务服务业、软件与信息服务业营业收入分别增长33.3%和85.8%，宁波广告产业园成为国家级广告产业园，人力资源服务机构数位居全省第一。现代绿色农业发展步伐加大。粮食和经济作物实现种植面积、产量“双增”，一产增加值314.1亿元，增长2.4%。新增省级现代农业产业园区4个、省级特色农业强镇6个，建成多彩农业美丽田园示范基地20个，慈溪现代农业产业园入选国家现代农业产业园创建名单。新增省级骨干农业龙头企业9家、市级示范性农民专业合作社和家庭农场45家。农村土地流转率和规模经营率均居全省第一。

　　（二）科技创新和质量效益得到新提升。创新转型步伐加快。R&D经费占比2.35%，规上工业企业科技活动经费支出增长21.7%。浙东南国家自主创新示范区创建扎实推进，获批国家海洋经济创新发展示范城市，鄞州区和中科院宁波材料所成为国家级“双创”示范基地。新增省级军民融合产业示范基地1家、省级以上众创空间13家、院士工作站16家、博士后工作站20家、高新技术企业716家，创新型初创企业突破1万家。发明专利授权量达到5400件，超级电容器项目获中国专利金奖。实施“3315”“泛3315”计划，新引进全职院士2名，海外高层次人才突破1万人。“质优宁波”建设扎实推进。开展全面质量提升行动，联动推进“三强一制造”，制（修）订国际（国家）标准49项，新增“浙江制造”品牌认证产品35项，宁波文具产业和海曙时尚纺织服装产业获批全国产业集群区域品牌建设试点，建成国家级出口产品质量安全示范区5个，食品和农产品抽检合格率分别达到96.7%和98.5%。企业经济效益稳步增长。规上工业企业、服务业企业利润分别增长30.9%和20%，规上工业单位增加值能耗下降4.0%，国有企业资产总额突破1万亿元。

　　（三）改革开放活力得到新提升。推进“最多跑一次”改革。狠抓“一窗受理、集成服务”，市县两级行政审批类“最多跑一次”事项占比分别达到89%和93.6%，1/3以上行政审批事项实现“全城通办”。8个功能园区推广“打包审批、集成服务”，平均减少行政审批承诺时限22个工作日。市县自建系统“信息孤岛”基本打通，154个乡镇（街道）“四个平台”建成运行。加快重点领域改革步伐。推进旅游体制改革，调整设立市旅游发展委员会，完善工作机制。改革招商引资体制，调整设立市经济合作与投资促进局，成立中东欧博览与合作事务局。109项政府职能转由社会组织承接，438家行业协会商会与行政机关脱钩。市属国有企业功能分类全面完成。打造优质营商环境，小微企业贷款余额突破5000亿元，制造业贷款同比多增374.6亿元，全年减免各类税费435.8亿元，稳妥处置特困企业44家。推行耕地保护补偿制度，启动国有建设用地二级市场改革试点，划定永久基本农田276.7万亩，盘活存量土地2.9万亩。农村承包地确权登记颁证基本完成，农房确权登记试点有序启动，江北区获批全国农村集体产权制度改革试点。开展教师“县管校聘”试点，跨省异地就医住院费用直接结算工作处于全省前列，成为首批国家级公立医院综合改革示范城市。促进开放型经济优化升级。坚持外贸优进优出发展，出口额占全国比重达到3.25%。国际邮件交换站开通运营，跨境电商交易额超过90亿美元，“双十一”跨境电商进口零售交易单量、交易总额均居全国第一。成立甬商总会，举办甬港经济合作论坛、海内外宁波周和中东欧国家投资贸易博览会、中东欧国家合作发展论坛、中国航海日论坛、国际港口管理机构圆桌会议，发布海上丝路贸易指数。新增备案（核准）境外中方投资39亿美元，国内招商引资实到资金突破1000亿元。获批中国—中东欧国家贸易便利化国检试验区，宁波南部滨海新区成为省级经济开发区。深化区域合作交流。积极融入长江经济带建设，主动参与长三角区域和浙东五市合作，对口支援帮扶和“山海协作”工程取得新成效。

　　（四）城乡融合水平得到新提升。优化区域开发布局。启动编制新一轮城市总体规划，开展东钱湖区域和“三江六岸”优化发展研究，推进四明山、象山港等区域保护开发。加快沪嘉甬铁路等项目前期工作。提高城市建设品质。启动实施国家城市设计、“城市双修”试点，开工建设海绵城市项目166个、地下综合管廊10.1公里。东鼓道地铁商业街等项目建成开业。新改建和整治城区主干道156公里，整治城市河道205条、316.6公里，新增城市公园23个、公共绿地336万平方米，中心城区生活垃圾分类覆盖率达到82%。改造棚户区446万平方米，三年目标任务圆满完成。完善城乡基础设施。梅山红桥、世纪大道一期高架主线通车，金甬铁路、轨道交通、三官堂大桥加快建设，杭甬高速复线宁波段一期、沈海高速石浦连接线动工。宁波至杭州湾新区引水工程启动，水库群联网联调（西线）一期工程贯通。新增污水管网1300多公里。改善城乡发展面貌。改造旧住宅区、旧厂区、城中村3695万平方米，拆除违法建筑2227万平方米，城镇危旧房屋三年治理改造任务全面完成。小城镇环境综合整治扎实开展，慈城镇、梁弄镇、西店镇成为全国特色小镇，9个镇入选省小城市培育试点三年行动计划名单，创建国家级美丽宜居示范村3个、省级美丽乡村示范镇（乡）11个、精品特色村31个。宁海县成为省级美丽乡村示范县，鄞州区、象山县成为省级“四好农村路”示范县，余姚市成为全国文明城市。

　　（五）群众生活品质得到新提升。促进就业增收。城镇新增就业18.5万人，发放就业援助补贴6.5亿元，城镇登记失业率2%。年收入6000元以下人群全部消除。家庭人均年收入6000元以下的现象全部消除。繁荣文体事业。广播剧《呦呦青蒿》入选全国“五个一工程”，10部文艺精品入选省“五个一工程”，“一人一艺”全民艺术普及、全民阅读推广工程深入开展，建成农村文化礼堂125家。成功举办亚洲艺术节、中国合唱节、阳明文化周、宁波国际赛车赛，上林湖越窑遗址公园成为国家考古遗址公园，全省首个“中国关心下一代教育示范基地”落户奉化。我市运动员在第十三届全运会上创造11金13银12铜的历史最好成绩。狠抓生态文明。城区空气优良率达到85.2%，地表水环境质量优良率达到71.3%，新划定生态保护红线面积1687.4平方公里，成为全国首批水生态文明城市，象山县成为首批国家生态文明建设示范县。淘汰改造高污染燃料锅炉1038台，县控以上劣Ⅴ类水质断面全面消除，劣Ⅴ类小微水体基本消除，34个乡镇建成“污水零直排区”，城镇集中式污水处理厂提标改造基本完成。查处环境违法行为2135起，中央环保督察组交办的信访件全部办结。发展教育事业。新改扩建幼儿园68所，公办和普惠性幼儿园覆盖面达到85%，改造义务教育段薄弱学校34所，率先实现所有区县（市）通过省教育基本现代化评估。3所高职院校入选省高职重点、优质建设学校名单。推进中国科学院大学宁波材料工程学院建设，启动宁波大学科技学院迁建工程，挂牌成立老年大学30所。完善保障体系。启动居家和社区养老服务改革试点、长期护理保险制度试点，户籍人口基本养老保险参保率、基本医疗保险参保率分别位居全省第一和第二，养老机构医疗卫生服务覆盖率达到81%，退休人员养老保障待遇稳步提高。低保边缘家庭救助制度基本建立，困难残疾人生活补贴和重度残疾人护理补贴实现全覆盖。建成保障性安居住房4.6万套，房地产市场平稳健康发展。保障群众健康。制订《健康宁波2030行动纲要》，深入实施“双下沉、两提升”，市级医院托管县级医院、县级医院托管乡镇卫生院实现全覆盖，新增床位2027张、执业医师1145名，家庭医生签约服务重点人群达到76万人，宁波“云医院”获信息社会世界峰会大奖。全民健身活动蓬勃开展，食品药品安全“两网五体系”工程全面实施。加强平安建设。圆满完成党的十九大维稳安保任务。实施新型居住证制度，强化社会治安管理，刑事案件立案数下降24.4%。加强互联网金融风险整治，严厉打击恶意逃废债行为，不良贷款率下降0.82个百分点。做好防台防汛工作，综合治理地质灾害83处。生产安全事故起数、死亡人数分别下降16.8%和20.8%。海曙区、江北区和宁海县获得“平安金鼎”。

　　一年来，我们始终坚持正确的政治方向，把忠诚核心、拥戴核心、维护核心作为最大的政治，牢固树立“四个意识”，深入推进“两学一做”学习教育常态化制度化，切实加强政府自身建设。坚持依法行政。自觉接受人大、政协和社会各界监督，建立市政府常务会议研究建议提案办理机制，办理代表建议609件、委员提案575件，提请市人大常委会审议地方性法规5件。修订市政府工作规则，完善重大事项集体决策机制和政府性债务严格管理制度，完成重大行政执法决定法制审核国家级试点工作。制定政府规章9件，审查行政规范性文件494件，办理行政复议案件1033件，公开行政处罚结果27.3万件。注重工作实效。开展“大脚板走一线、小分队破难题”及“打通中梗阻、提升执行力”等抓落实专项行动，完善经济运行监测“双底线”管理，行政审批权力事项提前办结率达到98.6%，12345政务热线服务满意率达到97%。严格廉洁从政。全面落实政府党组主体责任，持续推进廉政建设和作风建设，深化领导干部经济责任审计，“三公”经费支出下降5.9%。

　　一年来，我们支持国防和军队建设，加强国家安全、人民防空、民族宗教、应急管理、优抚安置等工作，哲学社会科学、档案史志、老龄、妇女儿童、慈善、红十字、外事、侨务、港澳台等工作实现新的发展。

　　各位代表！过去的一年，取得这样的成绩来之不易。这是党中央国务院、省委省政府和市委正确领导、科学决策的结果，是全市上下团结奋斗、顽强拼搏的结果。在这里，我代表市人民政府，向全市人民，向市人大代表、市政协委员，向离退休干部，向民主党派、工商联、人民团体和社会各界人士，向驻甬人民解放军和武警部队官兵，向驻甬部省属机构，向“宁波帮”和帮宁波人士，向所有关心支持宁波发展的海内外朋友们，表示衷心的感谢！

　　同时，我们也清醒地看到，发展中还存在一些不平衡不充分的问题：传统产业改造提升不够快，新经济培育不够充分，现代服务业发展还有很大空间；科技创新、管理创新、模式创新等方面还比较薄弱，高端人才、专业人才比较紧缺；固定资产投资增速放缓；城市集聚辐射能力有待增强，城乡建设发展品质有待提升，城乡区域发展还不够平衡；生态环境保护和治理任务依然艰巨，民生领域还有不少短板，教育、医疗、养老、交通等公共服务有待加强，影响社会和谐稳定的风险点还有不少。一些政府工作人员的服务能力、担当意识还不够强，形式主义、官僚主义等“四风”问题仍一定程度存在。对此，我们将高度重视，坚持问题导向、目标导向、实效导向，以更努力的工作、更扎实的举措，切实加以改进。

　　二、2018年主要目标任务

　　2018年是贯彻落实党的十九大精神的开局之年，是改革开放40周年，也是我市决胜高水平全面建成小康社会、实施“十三五”规划承上启下的关键一年。做好2018年工作，意义十分重大。

　　2018年政府工作的总体要求是：高举习近平新时代中国特色社会主义思想伟大旗帜，全面贯彻党的十九大、中央经济工作会议和省第十四次党代会、省委十四届二次全会、省委经济工作会议精神，认真落实市第十三次党代会、市委十三届三次全会和市委经济工作会议的决策部署，坚持稳中求进工作总基调，坚持新发展理念，紧扣社会主要矛盾变化，按照高质量发展的要求，统筹推进“五位一体”总体布局和协调推进“四个全面”战略布局，坚定不移沿着“八八战略”指引的路子走下去，大力弘扬红船精神，秉持浙江精神，传承宁波精神，突出“改革强市、创新强市、开放强市、人才强市”导向，以供给侧结构性改革为主线，加快推动质量变革、效率变革、动力变革，全面抓好改革开放、增长转型、改善民生、整治环境、防范风险、维护稳定各项工作，赶超发展，争先进位，把“名城名都”建设全面推向新境界，为浙江“两个高水平”建设作出更大贡献。

　　综合考虑发展趋势和工作导向，建议2018年经济社会发展的主要预期目标为：地区生产总值增长7.5%左右，财政收入、城乡居民收入增长与经济增长基本同步，居民消费价格涨幅3%左右，城镇新增就业15万人，城镇调查失业率、登记失业率分别控制在5%和3%以内，货物出口总额增长8%，R＆D经费占比2.5%，全员劳动生产率增长8%，单位生产总值能耗和二氧化碳、污染物排放量确保完成省里下达目标。在实际工作中，我们努力争取更好的结果。

　　围绕上述总体要求和预期目标，我们将拉高标杆创一流、扬长补短创优势，重点抓好九个方面的工作：

　　（一）着力筑牢发展根基，增强实体经济竞争力。实体经济是国民经济的命脉，是一个城市综合实力的重要体现，也是赶超发展的主要引擎。我们必须咬定青山不放松、久久为功抓实业，全力推进高质量发展。

　　大力发展先进制造业。积极争创“中国制造2025”国家级示范区，规上工业增加值增长8.5%。把数字经济作为“一号工程”来抓，聚焦智能制造、智能城市、智能港航等重点领域，推动实体经济与互联网、大数据、人工智能深度融合。培育发展智能终端产业，建设宁波杭州湾新区智能终端产业园，举办全球智能经济峰会、中国机器人峰会，争取成为国家5G试点城市。推进战略性新兴产业倍增发展，继续实施规上工业企业技术改造、智能化诊断三年行动计划，加快打造一批千亿级产业集群。大力培育企业梯队，扶持7家千亿级龙头培育企业、94家行业骨干培育企业、100家高成长培育企业、162家单项冠军培育企业，滚动实施“小微企业三年成长计划”。推进“凤凰行动”宁波计划，新增上市公司10家。

　　加快发展现代服务业。做大做强港航服务业，建设海上丝路航运大数据中心，推动宁波舟山港成为国际一流的现代化枢纽港。积极培育新兴业态，发展平台经济、分享经济、体验经济、创意经济。全面实施旅游发展五年行动计划，构建全域旅游联动体制机制，建设国际性休闲旅游目的地，旅游业增加值占地区生产总值比重达到7%。改造提升批发零售业，创新消费促进政策，推进网络市场监管与服务示范区建设，社会消费品零售总额增长10%。壮大总部经济，培育会展经济，提升发展科技信息、检验检测、法律咨询等中介服务业。加大金融支持实体经济力度，深化普惠金融综合示范区、科技与金融结合试点城市建设，发展股权融资、债券融资，完善“政银担”风险分担机制，人民币存贷款余额分别增长5%和7%。加快国家保险创新综合试验区建设，积极引进保险法人机构，拓展保险产品种类，培育保险产业基地，打造国内航运保险创新中心。

　　抢抓好项目大项目。坚持“一把手”招商不动摇，实施市、区县（市）长项目工程，实际利用外资43亿美元，浙商回归实到资金800亿元。强化区域统筹招商，突出招强引优、以商引商、产业链招商，更好发挥“宁波帮”和各级商会、协会的作用，提升全球化、市场化、专业化招商水平。谋划宁波杭州湾滨海新城产城联动二期等一批项目，开工吉利纯电动汽车等一批项目，推进中芯国际集成电路、中交未来城等一批项目，投产拓普150万套智能刹车系统等一批项目。工业投资增长8%，高新技术产业投资、民间投资均增长10%以上。

　　（二）着力推动创新发展，促进新旧动能转换。创新是引领发展的第一动力，人才是支撑发展的第一资源。我们将以更强的决心、更大的力度、更活的机制，加快建设国家创新型城市。

　　推进创新平台建设。加快建设浙东南国家自主创新示范区，大力提升宁波新材料科技城、国际海洋生态科技城、航天智慧科技城、中官路创业创新大街、南北高教园区和国家级“双创”示范基地的发展能级。实施“名校名院名所名人”引进工程，推动中科院宁波材料所、兵科院宁波分院、清华长三角研究院宁波分院加快发展，支持吉利沃尔沃汽车研究院、万华宁波高性能材料研究院等产业技术研究机构建设，建成国家智能制造装备质检中心、国家磁性材料计量测试中心，促进石墨烯创新中心创建国家级创新中心。加强小微企业创业创新基地建设，培育一批众创空间和公共研发平台，创建宁波新材料产业创新服务综合体。推动军工技术与制造业有机融合、军民科技成果双向转移转化，积极创建国家军民融合创新示范区。

　　完善创新发展环境。强化以企业为主体的技术创新体系建设，集成创新要素资源，推动产业链协同创新和产业孵化集聚创新。实施8个“科技创新2025”重大专项，推进国家科技成果转移转化示范区建设，设立科技成果转化引导子基金，发挥天使投资引导基金等作用。深化国家知识产权区域布局试点，优化知识产权运营服务体系，建设中国（宁波）知识产权保护中心。支持企业开展跨国技术并购，集聚全球高端创新资源。弘扬劳模精神、工匠精神和甬商精神，激发和保护企业家精神，让崇尚创新、宽容失败的社会氛围更加浓厚。

　　强化创新人才支撑。千方百计抢人才，制定人才强市行动纲要，大力实施“3315”“泛3315”和“资本引才”计划，加快引进一批海内外高精尖人才。打造国家海外人才离岸创新创业基地等重大引智平台。实施“甬智回归工程”和青年才俊储备专项行动，举办首届中国（宁波）海外工程师峰会，建设一批人才联络服务中心，争创国家级人力资源服务产业园区。大力培养本土人才，创新人才引进机制，积极吸纳高校毕业生就业创业，人才总量突破235万名。

　　（三）着力推进全面深化改革，营造一流的制度环境。改革是推进高质量发展、争创发展新优势的关键一招。我们必须坚定不移高举改革大旗，争当改革排头兵，勇做改革先行者。

　　深化“最多跑一次”改革。整合信息系统，破解信息孤岛，建设公共数据大平台，加快事项全面覆盖、投资审批全面覆盖、流程全面优化、乡镇（街道）全面延伸、标准全面统一，努力实现“快速办、就近办、网上办、移动办、全城通办”。推进受理与办理、办理与监督评价相分离，深化跨部门、跨层级“一件事情”梳理归集，拓展“一事联办”范围，减事项、减次数、减材料、减时间，在商事登记、不动产登记、企业投资项目、人才服务等领域取得新突破。推广“标准地”制度，确保一般企业投资项目开工前全流程审批100天内完成。推行“双随机、一公开”，加强事中事后监管，做到审批更简、监管更强、服务更优。

　　深化供给侧结构性改革。引导企业创新管理模式，建立健全现代企业制度。加快国有经济布局优化、结构调整和战略重组，推进竞争类国有企业混合所有制改革。提高供给体系质量和效率，加大“质优宁波”建设力度，支持企业实施“互联网+”“标准化+”“品牌+”等行动。加快破除无效供给，严格执行环保、能耗、技术、质量、安全等法规标准，有效处置“僵尸企业”，淘汰落后产能企业100家。深化要素配置市场化改革，强化“亩产论英雄”导向，促进生产要素流向优质高效领域。推进投融资创新，探索“XOD+PPP”模式，培育“基础设施+产业+生态”建设发展模式。深化国有建设用地二级市场改革试点，加快盘活利用存量土地。加大企业降本减负力度，进一步清理涉企收费，加强垄断行业价格监管和成本监审，降低企业制度性交易成本和融资、用能、用电、用水等生产要素成本。完善市场准入负面清单，健全社会信用体系，进一步激发企业创业创新活力。

　　（四）着力融入“一带一路”建设，形成全面开放新格局。开放是宁波的最大优势，也是繁荣发展的必由之路。我们要以“一带一路”建设为统领，全面提升新一轮对外开放水平。

　　打造重大开放平台。积极创建国家“一带一路”建设综合试验区，全力申报自由贸易港。建设“16+1”经贸合作示范区，深化中国—中东欧国家贸易便利化国检试验区建设，办好中国—中东欧国家经贸促进部长级会议，推动中东欧投资贸易博览会成为国家级展会，努力打造中东欧商品进口、投资合作和人文交流首选地。加快自贸区政策复制落地，大力推进重点开发区和功能区创新转型、提升发展。

　　巩固外贸强市地位。拓展欧美日主要市场和“一带一路”等新兴市场，建设境外营销网络和公共服务平台，外贸出口保持全国份额。深入转变外贸发展方式，服务贸易总额增长10%。实施新一轮“外贸实力效益工程”，推进“万企贸易成长计划”，培育消费品内外销“同线同标同质”企业，深化国家外贸综合服务企业试点。积极扩大进口，打造区域性特色进口商品基地和进口仓储物流集散地。深化“三互”大通关建设，完善国际贸易“单一窗口”功能，进一步提升贸易便利化水平。建设全国跨境电商中心城市，跨境电商交易额突破150亿美元。

　　深化内外交流合作。建好中意、中捷等国别园区，推进“侨梦苑”建设。有效引导支持对外投资，在中东欧、东南亚等地合作新建一批境外经贸合作区。扩大国际友城“朋友圈”，发挥索非亚中国文化中心等纽带作用，继续办好甬港经济合作论坛、海丝港口国际合作论坛、海内外宁波周和浙洽会、消博会。主动融入长江经济带、长三角一体化建设，深化浙东五市经济合作，扎实开展对口支援帮扶和“山海协作”工程。

　　（五）着力推进“四大建设”，加快构建现代大都市。建设大湾区、大花园、大通道、大都市区是省委、省政府作出的重大战略部署，我们必须抢抓机遇，积极推进。

　　打造大湾区中心城市。编制湾区经济发展规划和实施方案，整合宁波杭州湾新区、慈溪产业新城、余姚工业园等平台，建设环湾智能经济新区。主动承接上海城市功能和产业转移，深化沪甬合作示范区建设。争创国家临空经济示范区，支持宁波南部滨海新区、象山大目湾新城、宁南贸易物流园区特色发展。加快月湖金汇小镇、四明金融小镇、智能汽车小镇、芯港小镇等特色小镇创新发展。

　　打造大花园美丽城市。推进四明山区域生态修复，加强象山港区域保护利用，持续提升“三江六岸”品质。建设“美丽公路”精品线560公里、“美丽航道”50公里、“美丽绿道”140公里。创建天一阁·月湖、象山影视城5A级景区，启用梅山万人沙滩，建设宁波滨海旅游休闲区、华强二期等项目。综合整治城乡环境，开展“围挡”整治专项行动，“三改”和拆违各1000万平方米，实现“无违建创建先进区县（市）”全覆盖。加强城市管理，提高科学化、精细化、智能化水平。实施“厕所革命”三年行动计划，建设特色花卉景观大道，中心城区新增公共绿地100万平方米，使城市环境更加清爽、更加宜人。

　　打造大通道枢纽城市。加快交通强市建设，谋划集空港、高铁、地铁、地面交通于一体的综合交通枢纽。全力推进沪嘉甬铁路项目，力争列入国家“十三五”综合交通发展规划实施类项目。推进金甬铁路、杭甬高速复线宁波段一期、象山港疏港高速一期、梅山港区集装箱码头等项目，建成三门湾大桥及接线、杭州湾跨海大桥杭甬高速连接线等项目。深化余慈中心连接线等项目前期，谋划甬舟铁路、杭甬智能高速公路和杭甬、甬台温城际铁路等项目。协同加快义甬舟开放大通道、舟山江海联运服务中心建设。推进栎社国际机场三期项目，提升空港发展能级，机场旅客吞吐量突破1000万人次。

　　打造大都市区核心城市。编制新一轮城市总体规划，深化“多规融合”，优化“拥江揽湖滨海”空间布局。继续做好行政区划调整后续工作。推动中心城区“东集聚、南融合、西开发、北提升”，优化提升三江口片区，加快建设东部新城、南部新城、镇海新城、两江北岸等区域，高水平谋划推进东钱湖区域发展，统筹开发西部区块。深化城市主干道（街区）综合整治。开工建设环城南路西延二期、东钱湖快速路等项目，加快建设中兴大桥、三官堂大桥等跨江桥梁。推进轨道交通2号线二期、3号线一期、4号线、5号线一期和宁奉城际铁路建设。实现钦寸水库引水工程全线通水。加快城市有机更新，深化国家城市设计、“城市双修”试点，推进国家海绵城市建设。实施全国老旧小区改造试点，提高物业管理水平，稳妥有序开展既有多层住宅加装电梯。建设装配式建筑示范城市，推动建筑业提升发展。高标准建成国家公交都市示范城市，改善路网交通管理，切实缓解交通拥堵。

　　（六）着力实施乡村振兴战略，谱写美丽乡村新篇章。实施乡村振兴战略是新时代“三农”工作的总抓手。我们将加快推动农业全面升级、农村全面进步、农民全面发展，创建城乡融合发展先行示范区。

　　加快现代农业发展。坚持质量兴农、绿色兴农，深化农业供给侧结构性改革。稳定粮食综合生产能力，发展现代种业，确保粮食播种面积190万亩、蔬菜播种面积135万亩，生猪饲养量稳定在150万头以上。推进渔业提质增效发展。建设绿色都市农业示范区，创建4个省级以上现代农业园区、5个省级特色农业强镇、3条5亿级以上农业全产业链、20个多彩农业美丽田园示范基地。做精特色优势产业，实施农业绿色发展七大行动，增加优质农产品供给。培育现代农业经营主体，新增市级农业龙头企业、示范性农民专业合作社和家庭农场共45家。强化农村一二三产融合，发展乡村旅游、休闲农渔业，培育“农业+”“民宿+”等新业态，民宿经济收入增长25%以上。

　　提升农村生活品质。加强村庄规划设计，推进美丽乡村建设，市级示范村、示范乡镇累计分别达到180个、60个，风景线、A级景区村庄累计分别达到40条、350个。农村生活垃圾分类处理覆盖率达到50%。狠抓小城镇环境综合整治，确保50个乡镇（街道）通过省级考核验收。深入实施“防洪排涝2020行动计划”，开工建设东钱湖北排、鄞江堤防等工程，推进姚江二通道（慈江）建设，加快小流域治理、山塘整治和乡镇、村庄水环境示范整治。健全自治、法治、德治相结合的乡村治理体系，创建农村文明示范线，弘扬优秀乡土文化，发挥文化礼堂主阵地作用，推动形成文明乡风、良好家风、淳朴民风。

　　推进涉农体制机制改革。健全城乡融合发展体制机制和政策体系，深化农村产权制度、粮食收储制度和“三位一体”农合联改革，推进农村土地“三权分置”和林地制度改革试点，加快农村不动产统一登记制度改革，农房登记率达到70%以上。探索农村土地整村整镇流转，延长土地经营权流转周期，扩大规模经营面积。完善农业生产、经营和社会化服务体系，扩大涉农保险和信贷覆盖面。创新村级集体经济发展方式，探索农村“三资”“四制”发展模式，全面消除集体经济收入20万元以下的村。按照精准脱贫要求，加大帮扶力度，低收入农户收入增长10%以上。

　　（七）着力推动文化繁荣兴盛，提升城市发展软实力。文化是城市的灵魂，是人民的精神家园。我们要加快打造文化强市，为“名城名都”建设构筑强大精神力量。

　　深化文明城市建设。大力宣传习近平新时代中国特色社会主义思想，培育和践行社会主义核心价值观，深化中国梦主题宣传教育，推进社会公德、职业道德、家庭美德、个人品德建设。开展移风易俗、弘扬时代新风行动，加强科学普及工作。开展新一轮文明城市创建，提升市民文明素养和全社会文明程度，建设全域化高水平文明城市。深化拓展未成年人思想道德建设，力争市县两级文明校园比例分别达到30%和70%。培育壮大志愿者队伍，注册志愿者达到180万人。

　　完善公共文化服务。加强优质公共文化产品和服务供给，建设城市文化公园、社区文化家园，完善文化馆总分馆制，投用市图书馆新馆。加强文化传承创新，挖掘弘扬浙东文化、商帮文化、阳明文化，抓好文物保护利用和文化遗产保护传承，加快雪窦山佛教五大名山建设。组织艺术精品创作，打响宁波交响乐团品牌，建设全国“一人一艺”全民艺术普及先行区。

　　加快文化产业发展。推进文化创意街区建设，做大做强影视城、文创港、音乐港、时尚港等文化产业平台，深化国家文化消费城市试点，举办文化消费季活动，培育新型文化业态和文化消费品牌。创建国家文化与金融合作示范区，创新文化金融服务产品。加强政策引导，扶持一批基础较好、实力较强的文化企业。文创产业增加值占地区生产总值比重提高到7%。

　　（八）着力加强环境治理，提高生态文明水平。绿水青山就是金山银山。我们要深入践行“两山”重要思想，坚决打好污染防治攻坚战。

　　实施“碧水蓝天净土”工程。巩固治水剿劣成果，深化“河长制”，推行“湾（滩）长制”“湖长制”，开展饮用水源地环境保护行动，深入推进乡镇（街道）“污水零直排区”建设，确保劣Ⅴ类水无新增、不反弹。加强海洋环境保护，治理近岸海域漂浮垃圾。强化“五气共治”，PM2.5年均浓度降至37微克/立方米以下，城区空气优良率超过86%。防治土壤污染和固体废弃物污染，整治废弃矿山生态环境。抓好中央环保督察和国家海洋督察反馈问题整改，坚决制止和严厉惩处破坏生态环境行为。

　　推行绿色生产生活方式。发展循环经济，开展“绿色制造工程三年攻坚行动”，实施节能改造项目300个，节约标准煤50万吨。严控高耗能、高污染和资源性项目准入，推进“低小散”行业专项整治。推进屋顶光伏工程，优化电动汽车充电基础设施布局，加快宁海抽水蓄能电站建设。强化资源节约集约和综合利用，完善垃圾处理设施，中心城区生活垃圾分类处理覆盖率达到85%。

　　完善生态文明体制。强化国土空间用途管制和生态红线管理，深入做好土地调查。健全自然资源资产产权制度，推进自然资源统一确权登记，编制自然资源资产负债表，探索市场化、多元化生态补偿机制。推进碳排放权交易市场建设，完善用能权、排污权有偿交易和差别化价格机制，加快排污权核定与收储。开展领导干部自然资源资产离任审计，严肃追究生态环境损害责任。

　　（九）着力改善民生福祉，增强人民群众获得感。人民对美好生活的向往，是我们始终不渝的奋斗目标。我们要切实解决好人民最关心最直接最现实的利益问题，努力让人民生活更加幸福美满。

　　优先发展教育事业。提高教育现代化水平，协调推进学前教育、义务教育、高中段教育、职业教育、高等教育发展。尽力破解儿童早期教育服务问题，巩固公办和普惠性幼儿园覆盖面，新改扩建幼儿园40所，新增入学名额1万个。改造义务教育段薄弱学校20所。深化课程改革，推进高中教育特色化、多样化发展。加强社会培训机构治理，着力破解中小学生课外负担重等问题。深化与“一带一路”国家的职业教育合作。推进宁波大学一流学科高校、浙江大学宁波“五位一体”校区和中国科学院大学宁波材料工程学院建设，提高产教融合水平。加强特殊教育，实施终身教育提升工程。推进教师“县管校聘”改革，加强师德师风建设，培养高素质教师队伍。

　　提高就业创业水平。实施就业优先战略和积极就业政策，提供全方位公共就业服务，深化创业型区县（市）建设，拓宽居民劳动收入和财产性收入渠道。推进“技能宁波”三年行动计划，创建国家技能振兴综合示范区，加强职业技能培训，建设一批高技能人才公共实训基地、实习见习基地，培育高技能人才3万名。建设创业孵化基地，积极扶持青年创业。构建和谐劳动关系，打造劳动纠纷多元化解服务平台，大力创建“无欠薪”区县（市）。

　　完善社会保障体系。推进参保扩面，户籍人口基本养老保险参保率超过94.6%、基本医疗保险参保率超过98%，工伤保险实现职业人群全覆盖。健全大病保险制度，实施医保历年账户余额购买商业健康保险。完善社会救助、社会福利、慈善事业、优抚安置等制度，探索解决婴幼儿照护问题，保障妇女儿童权益，发展残疾人事业。坚决落实房子是用来住的、不是用来炒的定位，建立多主体供应、多渠道保障、租购并举的住房制度，启动新一轮棚改，推进住房保障货币化，促进房地产市场平稳健康发展。深化全国居家和社区养老服务改革、长期护理保险制度试点，加快建设区域性居家养老服务中心，养老机构医疗卫生服务覆盖率达到90%。

　　加快建设健康宁波。坚持“六医”统筹，实施《健康宁波2030行动纲要》。加强与知名医学院校和高端医院的合作、引进，培育名医名科名院。深化综合医改先行先试，创新互联网医疗服务应用，建设高水平医联体和县域医共体，提高家庭医生签约率和服务水平。建成宁波市杭州湾医院，开工市第一医院异地建设项目，加快医疗机构儿科建设。完善分级诊疗制度，基层医疗机构门急诊量占比超过65%。广泛开展全民健身活动，打造“一县一品”的品牌赛事体系，建成奥体中心“三馆一园”。创建国家食品安全示范城市、农产品质量安全市，加强放心农贸市场建设，完善食品药品安全全程追溯和治理体系，让市民吃得更放心。

　　构筑共建共治共享的社会治理格局。打好防范化解风险攻坚战，加强地方金融监管和风险监测预警，坚决打击违法违规金融活动，进一步降低不良贷款率，确保不发生区域性金融风险。加强政府性债务管理，确保风险可控制、财政可持续。开展“安全风险管控年”活动，推进地质灾害除险安居“清零行动”，加强智慧消防建设，深入整治公共安全风险隐患，坚决遏制重特大安全事故。推进“七五”普法，预防和化解社会矛盾，加强和改进信访工作，完善基层治理体系“四个平台”建设，打造新时期“枫桥经验”宁波样板。培育政府职能承接型、补位型社会组织，完善社区治理，促进社区、社会组织、社会工作“三社联动”。加强治安防控和应急处置能力建设，严厉打击各类违法犯罪活动，力夺“平安金鼎”。建立网络综合治理体系，防范和打击网上虚假信息诈骗、倒卖个人信息等行为。重视民族宗教工作。加强流动人口服务管理。推进国家安全、国防教育、国防动员、人民防空工作，争创省级双拥模范城“八连冠”。

　　各位代表！我们将始终坚持以人民为中心的发展思想，想群众之所想、急群众之所急、解群众之所困，全力以赴办好代表们确定的民生实事。

　　三、建设人民满意的服务型政府

　　新时代、新思想，新使命、新征程，对政府工作和自身建设提出了新的更高要求。我们将尽心尽力、尽职尽责，扎实推进“两强三提高”，不断提升政府施政能力和服务水平。

　　（一）坚定政治方向。牢固树立“四个意识”，增强“四个自信”，始终在思想上政治上行动上同以习近平同志为核心的党中央保持高度一致，坚决维护以习近平同志为核心的党中央权威和集中统一领导。深入学习宣传贯彻党的十九大精神，认真开展“不忘初心、牢记使命”主题教育，坚决把中央大政方针、省委省政府和市委的决策部署不折不扣落到实处。

　　（二）严格依法行政。深入建设法治政府，确保政府工作在法治轨道上运行。认真执行市人大及其常委会的决议决定，自觉接受市人大的法律监督和工作监督，主动接受市政协的民主监督，高质量办理建议提案。广泛听取各民主党派、工商联、无党派人士和人民团体意见，全面接受司法监督、群众监督和舆论监督，推进审计监督全覆盖。遵守法定权限和程序，严格规范公正文明执法，完成行政复议体制改革。发挥新型智库作用，推进依法、科学、民主决策。全面深化“五公开”，持续提升政府公信力。

　　（三）强化实干担当。营造崇尚实干、勇于担当的良好氛围，说实话、谋实事、出实招、求实效。大兴调研之风，深入基层一线，找准短板弱项，解决实际问题。大力推进公务人员专业化建设，增强专业素养、专业能力和专业精神。构建科学有效的考核机制，切实减轻基层负担。完善正向激励和容错纠错机制，让广大公务人员想干事、能干事、干成事。

　　（四）坚持从严治政。严格落实全面从严治党主体责任，深入贯彻中央八项规定及实施细则精神，坚持不懈纠“四风”、正作风。严厉查处损害群众利益的不正之风和腐败行为，构建亲清新型政商关系。坚持用制度管权管事管人，强化权力运行制约监督。完善财政预算管理制度，改进绩效管理，严格控制“三公”经费。完成事业单位、国有企业的公车改革。大力整治不作为、慢作为、乱作为，坚决打通“中梗阻”，不断提升执行力。

　　各位代表！实干托起梦想，奋斗铸就辉煌。让我们更加紧密地团结在以习近平同志为核心的党中央周围，高举习近平新时代中国特色社会主义思想伟大旗帜，在中共宁波市委的坚强领导下，不忘初心、牢记使命，勇立潮头、赶超发展，加快建设“名城名都”，为浙江“两个高水平”建设，为实现“两个一百年”奋斗目标而努力奋斗！
